# Integrating Ultrasonographic and Biochemical Markers to Assess Dengue Severity in Critical Care: A Retrospective Study

**DOI:** 10.7759/cureus.100016

**Published:** 2025-12-24

**Authors:** Shantanu Dey, Manish Gupta, Akash Mehrotra, Kuldeep Singh, Vishrant Shetty, Dipankar Dutta

**Affiliations:** 1 Critical Care Medicine, Max Super Speciality Hospital, Vaishali, Ghaziabad, IND

**Keywords:** dengue fever/complications, gallbladder wall thickness, icu outcome, severe dengue fever, transaminitis, ultrasound in critical care

## Abstract

Background and aims

Dengue fever can progress to severe disease with hepatic dysfunction and multiorgan involvement. Early identification of markers is essential for timely intervention. This study evaluated transaminitis and gallbladder wall thickness (GBWT) as markers of severe dengue in critically ill patients.

Materials and methods

A retrospective observational study was conducted on 120 serologically confirmed dengue patients admitted to a tertiary ICU between July and November 2023. Patients were categorized according to the WHO 2009 criteria into dengue with warning signs and severe dengue. Serum transaminase levels and GBWT (>5 mm) were recorded, and their associations with ICU stay, mechanical ventilation, dialysis, and 28-day mortality were analyzed.

Results

Severe dengue cases demonstrated significantly elevated serum glutamic oxaloacetic transaminase and serum glutamic pyruvic transaminase levels compared with patients presenting with warning signs (p < 0.001). GBWT >5 mm was observed in 75.93% of severe dengue cases versus 54.55% of warning-sign cases (p = 0.015). GBWT >5 mm was significantly associated with an increased need for mechanical ventilation (33.77% vs. 9.30%, p = 0.04), dialysis (23.38% vs. 6.98%, p = 0.025), and higher mortality (25.97% vs. 4.65%, p = 0.003).

Conclusion

Transaminitis and increased GBWT are simple, cost-effective, and clinically meaningful markers of severe dengue in critically ill patients. Early recognition of these parameters can support timely risk stratification and help guide appropriate ICU management strategies.

## Introduction

Dengue is one of the most important arthropod-borne viral infections worldwide, with a rapidly expanding geographic footprint and increasing incidence, particularly in tropical and subtropical regions, including India [1,2]. The World Health Organization (WHO) 2009 classification recognizes dengue with and without warning signs and severe dengue, reflecting the spectrum from self-limiting illness to life-threatening disease characterized by plasma leakage, bleeding, and organ dysfunction [3]. Despite advances in supportive care, severe dengue continues to contribute substantially to ICU admissions, healthcare resource utilization, and mortality in endemic countries [1-3].

Hepatic involvement is increasingly recognized as a key component of dengue pathophysiology. Liver injury may range from mild, asymptomatic transaminitis to acute liver failure and can coexist with other manifestations of severe plasma leakage and shock [4-6]. Elevation of aspartate aminotransferase (AST) and alanine aminotransferase (ALT) is common even in non-severe cases, but higher enzyme levels have been associated with dengue hemorrhagic fever, severe dengue, and adverse outcomes, leading the WHO 2009 guidelines to include AST/ALT ≥ 1000 U/L as one of the criteria for severe dengue [3,6]. Several reviews have described the wide spectrum of hepatic involvement in dengue and highlighted persistent uncertainty regarding the best way to use biochemical markers to risk-stratify patients [4,5].

In addition to biochemical markers, ultrasonography has emerged as a non-invasive tool to detect early plasma leakage. Gallbladder wall thickening is one of the most frequently reported sonographic findings in dengue and reflects increased capillary permeability [7,8]. Gallbladder wall thickness (GBWT) identifies subclinical plasma leakage through ultrasonographic markers such as gallbladder wall thickening, ascites, and pleural effusion, which may be present before classical clinical manifestations, such as hypotension, shock, or overt fluid accumulation, become apparent. Studies from Latin America and South Asia have demonstrated that GBWT > 3 mm is significantly associated with more severe forms of dengue and that marked thickening (>5-6 mm), often with a layered or “honeycomb” pattern, may identify patients at higher risk of developing hypovolemic shock, hemorrhagic manifestations, and ICU admission [7,8]. However, GBWT is not specific to dengue and may be influenced by fasting status and other comorbidities, so its integration with clinical and laboratory predictors remains an area of active research [7,8].

The concept of expanded dengue syndrome further underscores that dengue can affect multiple organ systems, including the liver, lungs, kidneys, and central nervous system, through a combination of viral cytopathic effects, immune-mediated injury, and microcirculatory dysfunction [9]. The elevation of transaminases in dengue is primarily due to hepatocellular injury, resulting from a combination of direct viral cytopathic effects, immune-mediated hepatocyte damage, and hepatic hypoperfusion related to microcirculatory dysfunction and plasma leakage. Additional contributors include cytokine-mediated inflammation and mitochondrial dysfunction, leading to hepatocyte membrane injury and the leakage of enzymes into the circulation. Within this broader context, simple, bedside-available markers such as transaminase levels and ultrasound findings may help clinicians identify patients who are likely to deteriorate and require critical care support. However, data specifically focusing on ICU-admitted adult dengue patients and the combined use of ultrasonographic (GBWT and other abdominal findings) and biochemical markers (transaminitis, albumin-globulin ratio, platelet count) to predict disease severity and ICU outcomes remain limited, especially from Indian tertiary care centers.

Therefore, we conducted a retrospective observational study of serologically confirmed adult dengue patients admitted to the ICU of a tertiary care hospital. Our primary objective was to evaluate the association between ultrasonographic findings, particularly GBWT, and biochemical markers of liver involvement with dengue severity and ICU outcomes. We sought to determine whether integrating these parameters could improve early risk stratification and guide timely, targeted management in critically ill dengue patients.

Preliminary findings from this study were previously presented as a poster at the Annual National Conference of the Indian Society of Critical Care Medicine and published as an abstract in the Indian Journal of Critical Care Medicine [10].

## Materials and methods

Study design

This was a retrospective, cross-sectional, observational study designed to evaluate the association between biochemical abnormalities, ultrasonographic findings, and clinical severity in patients diagnosed with dengue fever. The study adhered to the STROBE (Strengthening the Reporting of Observational Studies in Epidemiology) guidelines for observational research reporting. The Institutional Ethics Committee provided no objection and approval for the analysis and publication of de-identified retrospective patient data (IEC Ref. No.: BHR/ RS/MSSHNSH/CRUIEC/CCM/24-03), and the requirement for informed consent was waived.

Setting and study period

A retrospective observational study was conducted on 120 serologically confirmed dengue patients admitted to Max Super Speciality Hospital, Vaishali, a tertiary care center in North India with an overall capacity of more than 370 beds, between July and November 2023. All patient data, including clinical records, laboratory investigations, ultrasonography findings, treatment details, and outcomes, were collected from the hospital’s electronic medical record system. Cases were classified as dengue with warning signs or severe dengue, according to the WHO 2009 dengue guidelines [3].

Participants

Inclusion criteria included the following: adults aged 18 years and above, serologically confirmed dengue infection (positive NS1 antigen and/or IgM antibody), admission to the ICU during the study period, and availability of complete laboratory and ultrasonographic data. Exclusion criteria included pre-existing chronic liver disease, pregnancy, transfer from another facility with incomplete records, and alternative causes of transaminitis such as drug-induced, alcohol-related, or metabolic liver injury.

Cases were classified as dengue with warning signs or severe dengue, according to the WHO 2009 dengue guidelines [3]. These criteria define warning signs as abdominal pain or tenderness, persistent vomiting, clinical fluid accumulation, mucosal bleeding, lethargy or restlessness, liver enlargement >2 cm, and an increase in hematocrit with a concurrent fall in platelet count. Severe dengue is defined by severe plasma leakage leading to shock or respiratory distress, severe bleeding, or severe organ involvement such as AST/ALT ≥1000 IU/L or impaired consciousness.

Data collection and variables

Data extraction followed a standardized protocol and included demographics (age, sex), clinical parameters (duration of fever, hemodynamic status, organ dysfunction), biochemical parameters (AST, ALT, bilirubin, albumin, globulin, albumin-globulin ratio, hematocrit, platelet count), ultrasonographic parameters (GBWT, hepatomegaly, splenomegaly, ascites, pleural effusion), and outcome variables (need for oxygen therapy, non-invasive ventilation (NIV)/high-flow nasal cannula (HFNC), mechanical ventilation, vasopressor support, ICU length of stay, and mortality). Variables were recorded from the earliest available investigation set within the first 24 hours of ICU admission to maintain uniformity.

Operational definitions

Severe dengue was defined as per the WHO 2009 criteria, including severe bleeding, fluid accumulation leading to respiratory distress, shock, or organ impairment [3]. Transaminitis was defined as AST or ALT > 40 IU/L. Gallbladder wall thickening was defined as an ultrasonographic measurement ≥ 5 mm in this study, and plasma leakage was considered present when ascites and/or pleural effusion were noted on imaging.

Ultrasonography protocol

Bedside ultrasonography was performed by radiologists or trained intensivists using a high-frequency convex transducer. GBWT was measured in the fasting state whenever feasible. The first ultrasound performed after ICU admission was considered for analysis to reduce interobserver variability.

Outcome measures

The primary outcome was the association of biochemical and ultrasonographic parameters with dengue severity. Secondary outcomes included the need for organ support (NIV/HFNC, mechanical ventilation), vasopressor requirement, ICU length of stay, and in-hospital mortality.

Statistical analysis

Data analysis was performed using IBM SPSS Statistics for Windows, version 26.0 (IBM Corp., Armonk, NY). Continuous variables were expressed as mean ± standard deviation (SD) or median with interquartile range (IQR), depending on distribution, as assessed by the Shapiro-Wilk test. Categorical variables were presented as frequencies (N) and percentages (%). Comparisons between the severe and non-severe dengue groups were performed using the chi-square test or Fisher’s exact test for categorical variables and Student’s t-test or Mann-Whitney U test for continuous variables. All tables include numbers (%), a test statistic column, and p-values. A p-value of less than 0.05 was considered statistically significant.

## Results

**Figure 1:**
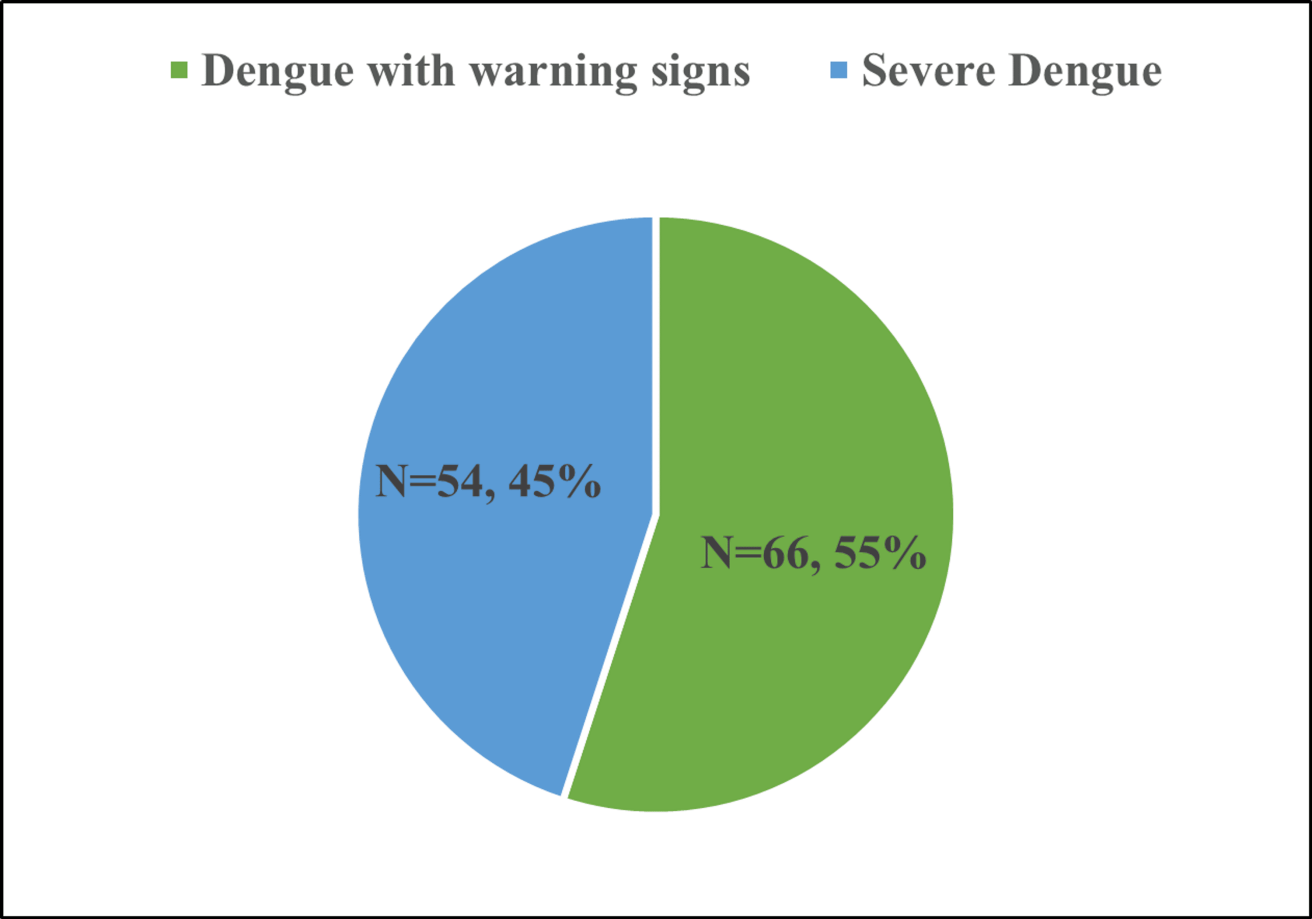
Distribution of patients according to dengue severity (warning signs vs. severe dengue). This figure depicts the proportional distribution of 120 ICU-admitted dengue patients classified using the WHO 2009 criteria. Of the total patients, 66 (55%) presented with dengue with warning signs, while 54 patients (45%) fulfilled criteria for severe dengue. The figure provides a visual overview of the disease burden and sets the context for subsequent comparisons of biochemical, ultrasonographic, and clinical outcome parameters between the two groups. No statistical test applies, as this represents descriptive categorization rather than group comparison.

A total of 120 serologically confirmed dengue patients admitted to the ICU during the study period were included in the analysis. The study population was divided into two groups based on disease severity: dengue with warning signs (55%) and severe dengue (45%) (Figure 1). The overall age range of patients was 15-80 years, with the mean age of patients with warning signs being 37.61 ± 14.07 years, and 37.96 ± 15.23 years in severe dengue patients. The proportion of males was higher than that of females in both groups; however, no statistically significant association with severity was observed. The demographic profile, serology, and presenting symptoms are summarized in Table 1.

**Table 1:** Demographic profile, dengue serology, and presenting symptoms in patients with dengue with warning signs versus severe dengue. This table summarizes baseline demographic characteristics, dengue serology patterns (NS1, IgM, IgG), and presenting clinical features among patients with dengue with warning signs versus severe dengue. Data include frequencies/percentages (N, %), along with statistical comparisons using chi-square or Fisher’s exact test. The table highlights symptoms significantly associated with severe dengue, such as vomiting, abdominal pain, respiratory distress, bleeding, shock, and multiorgan involvement, allowing early recognition of severe disease. * = p < 0.05; ** = p < 0.001. CVS: cardiovascular system; CNS: central nervous system.

Variable	Dengue with warning signs	Severe dengue	Test statistic	P-value
Age (years)	37.61 ± 14.07	37.96 ± 15.23	t = –0.13	0.894
Gender (female vs. male)	25.8%, n=17/74.2%, n=49	25.9%, n=14/74.1%, n=40	χ² = 0.0002	0.983
Dengue serology				
IgG	1.5%, n=1	0%, n=0	Fisher exact	0.83
IgG + IgM	3.0%, n=2	3.7%, n=2	χ² = 0.04	0.84
IgM	1.5%, n=1	1.9%, n=1	χ² = 0.02	0.88
NS1 antigen	93.9%, n=62	94.4%, n=51	χ² = 0.01	0.91
Presenting symptoms				
Fever	100%, n=66	100%, n=54	—	—
Headache	74.2%, n=49	81.5%, n=44	χ² = 0.89	0.345
Vomiting	74.2%, n=49	88.9%, n=48	χ² = 4.10	0.043*
Abdominal pain	74.2%, n=49	92.6%, n=50	χ² = 7.01	0.008*
Respiratory distress	6.1%, n=4	87.0%, n=47	χ² = 84.9	<0.001**
Bleeding	21.2%, n=14	72.2%, n=39	χ² = 40.3	<0.001**
Shock	0%, n=0	72.2%, n=39	Fisher exact	<0.001**
Polyserositis	53.0%, n=35	96.3%, n=52	χ² = 33.2	<0.001**
Myalgia	86.4%, n=57	94.4%, n=51	χ² = 2.15	0.142
CVS involvement	0%, n=0	74.1%, n=40	Fisher exact	<0.001**
CNS involvement	0%, n=0	59.3%, n=32	Fisher exact	<0.001**
Respiratory system involvement	10.6%, n=7	75.9%, n=41	χ² = 54.8	<0.001**
Per-abdomen involvement	27.3%, n=18	75.9%, n=41	χ² = 29.8	<0.001**

All patients were tested for dengue serology markers. All included patients had either the NS1 antigen and/or IgM positivity consistent with acute infection. In both groups, the majority were positive for NS1 antigen (93.90% and 94.40% in dengue with warning signs and severe dengue groups, respectively). Smaller proportions were positive for IgG, IgM, or both. The most common clinical presentation was fever (100% in both groups). A statistically significant difference between groups was noted for symptoms such as vomiting, abdominal pain, respiratory distress, bleeding, shock, polyserositis, and involvement of cardiovascular, central nervous, respiratory, and abdominal systems (Table 2).

**Table 2:** Outcomes in patients with normal gallbladder versus those with gallbladder wall thickening. This table compares outcomes (mechanical ventilation, dialysis, mortality, and ICU stay) between patients with normal gallbladder wall thickness (GBWT) and those with GBWT >5 mm. The findings underscore GBWT as an ultrasonographic marker of plasma leakage and a poor prognostic indicator. The statistical tests used include the chi-square/Fisher's test for categorical variables and the t-test/Mann–Whitney test for ICU stay. * = p < 0.05.

Variable	Normal GBWT (N=43)	GBWT >5 mm (N=77)	Test statistic	P-value
Need for mechanical ventilation	9.30% (N=39)	33.77% (N=51)	χ² = 8.28	0.004*
Need for dialysis	6.98% (N=40)	23.38% (N=59)	χ² = 5.03	0.025*
Discharged	95.35% (N=41)	74.03% (N=57)	χ² = 8.82	0.003*
Expired	4.65% (N=2)	25.97% (N=20)	χ² = 8.82	0.003*
ICU stay (days)	5.63 ± 4.42	5.01 ± 4.79	t = 1.01	0.311

Laboratory parameters are detailed in Table 3. Serum glutamic oxaloacetic transaminase (SGOT) levels were significantly higher in the severe dengue group (mean = 4383 ± 6845.07) compared with the warning signs group (mean = 254.73 ± 342.35), with p < 0.001. Serum glutamic pyruvic transaminase (SGPT) levels were likewise significantly higher in the severe dengue group (mean = 1280 ± 1670.08) compared with the warning signs group (mean = 156.11 ± 154.88), with p < 0.001.

**Table 3:** Comparison of laboratory parameters (SGOT and SGPT) and ICU stay between patients with warning signs and severe dengue. This table compares biochemical liver parameters (SGOT and SGPT) and ICU length of stay between patients with warning signs and those with severe dengue. Values are presented as mean ± SD, median (IQR), and observed ranges. Mann–Whitney U test or Student’s t-test was used based on data distribution. A significantly higher degree of transaminitis is seen in severe dengue, indicating hepatic involvement as a marker of severity. ** = p < 0.001. SGOT: serum glutamic oxaloacetic transaminase; SGPT: serum glutamic pyruvic transaminase.

Variable	Dengue with warning signs (n=66)	Severe dengue (n=54)	Test statistic	P-value
SGOT (U/L)	254.73 ± 342.35	4383 ± 6845.07	U = 674.0	<0.001**
SGPT (U/L)	156.11 ± 154.88	1280 ± 1670.08	U = 798.5	<0.001**
ICU stay (days)	4.65 ± 2.97	5.87 ± 6.06	t = –0.05	0.957

ICU stay was marginally longer in patients with severe dengue.

Abdominal ultrasonography revealed gallbladder wall edema in 54.55% of patients with warning signs and in 75.93% of patients with severe dengue (p = 0.015). As shown in Table 4, none of the patients with warning signs required dialysis or mechanical ventilation, whereas among those with severe dengue, 55.6% required mechanical ventilation and 38.9% required dialysis (p < 0.001 for both). A significant difference in 28-day mortality was also observed: 40.7% in the severe dengue group versus none in the warning-sign group (Table 4).

**Table 4:** Association of dengue severity with gallbladder wall thickness (GBWT), need for mechanical ventilation, dialysis, and 28-day mortality. This table demonstrates the relationship between dengue severity and key outcome variables, including gallbladder wall edema (>5 mm), need for mechanical ventilation, dialysis requirement, and 28-day mortality. Data are presented as percentages, and statistical significance was assessed using chi-square/Fisher tests. The table emphasizes that severe dengue is associated with markedly higher organ support requirements and mortality. * = p < 0.05; ** = p < 0.001.

Variable	Warning signs (n=66)	Severe dengue (n=54)	Test statistic	P-value
Gallbladder wall edema	54.55% (n=36)	75.93% (n=41)	χ² = 5.88	0.015*
Need for mechanical ventilation	0% (n=0)	55.6% (n=30)	Fisher exact	<0.001**
Need for dialysis	0% (n=0)	38.9% (n=21)	Fisher exact	<0.001**
Discharged	100% (n=66)	59.3% (n=32)	χ² = 27.8	<0.001**
Expired	0% (n=0)	40.7% (n=22)	χ² = 27.8	<0.001**

Outcomes were further evaluated based on GBWT. In patients with GBWT > 5 mm, a statistically significant association was observed with the need for mechanical ventilation (33.77% vs. 9.30%) and dialysis (23.38% vs. 6.98%). A markedly higher mortality rate was seen in patients with GBWT > 5 mm compared to those with a normal gallbladder wall (25.97% vs. 4.65%). These data are summarized in Table 4.

## Discussion

Dengue has become a global health concern, with increasing annual cases, particularly in tropical countries like India, where seasonal changes lead to spikes in infections [1,2]. Early diagnosis and disease stratification are essential for optimizing the prognosis and management of dengue patients. In the present study, we evaluated the utility of bedside parameters, specifically transaminitis and GBWT, as prognostic markers of dengue severity in critically ill patients admitted to a tertiary care ICU.

In our cohort of 120 confirmed dengue cases, 55% were classified as dengue with warning signs and 45% as severe dengue based on the WHO 2009 criteria [3]. This proportion of severe dengue is higher than that reported in some earlier general ward cohorts but is consistent with the higher acuity expected in ICU populations. The mean age and male predominance observed in both groups align with previous reports from endemic regions, suggesting that adult males may be more frequently exposed or more likely to present to hospital care.

Fever was the universal presenting symptom in our study, while other clinical features such as vomiting, abdominal pain, respiratory distress, bleeding manifestations, shock, and polyserositis were more common in the severe dengue group. These findings reflect widespread systemic involvement and plasma leakage in severe disease and underscore the importance of vigilant clinical monitoring for warning signs and organ system involvement in suspected dengue cases, particularly in the ICU setting.

Our findings are consistent with prior work showing that hepatic involvement is almost universal in hospitalized dengue patients and may serve as an early prognostic marker of disease severity [4-6]. Similar to observations from other cohorts, we found that patients with higher AST and ALT values were more likely to have severe dengue and required closer hemodynamic monitoring and organ support [6]. Reviews by Samanta and Sharma and by Dissanayake and Seneviratne likewise emphasize that dengue-related liver injury arises from a combination of direct viral cytopathic effects, immune-mediated damage, and hypoxic insult during shock, and that marked transaminitis should prompt careful assessment for impending decompensation [4,5].

The strong association of increased GBWT with severe dengue and adverse clinical outcomes in our cohort aligns with the growing body of literature on GBWT as an ultrasonographic marker of plasma leakage [7,8]. Tavares et al. showed that GBWT > 3 mm, particularly in the presence of cavitary effusions, was independently associated with severe dengue under the WHO 2009 classification [7]. Adil et al. further demonstrated that GBWT thresholds around 3.5-4 mm have high sensitivity and specificity for dengue hemorrhagic fever and can complement hematological parameters such as platelet count and hematocrit in risk stratification [8]. Our results extend these observations to an ICU population, suggesting that routine bedside ultrasound, including assessment of GBWT, can add important prognostic information beyond standard laboratory measurements.

Finally, the spectrum of hepatic and ultrasonographic changes in our patients supports the broader concept of expanded dengue syndrome, in which multiorgan involvement, including significant liver injury, may occur even in the absence of classic hemorrhagic manifestations [9]. Recognizing these atypical or extrapulmonary presentations is crucial in the ICU setting, where coexisting sepsis, shock, and organ dysfunction from other causes may obscure the underlying dengue-related pathology [1,9].

This study has several limitations. First, it was a single-center retrospective analysis with a relatively small sample size, which may limit the generalizability of the findings and preclude robust multivariable adjustment for all potential confounders. Second, serial measurements of liver enzymes and GBWT were not available for all patients, so we could not fully characterize the temporal evolution of transaminitis and ultrasonographic changes throughout the course of illness. Third, potential interoperator variability in gallbladder ultrasonography may influence the measurement of GBWT. Additionally, we acknowledge that the study did not capture the exact timing of organ support interventions (mechanical ventilation, intermittent hemodialysis/continuous renal replacement therapy) relative to liver function test and ultrasonography.

## Conclusions

This study highlights that markedly elevated transaminases and increased GBWT are strongly associated with severe dengue and adverse ICU outcomes. These readily available biochemical and ultrasonographic parameters can serve as practical tools for early risk stratification in critically ill dengue patients. Incorporating assessment of liver enzymes and GBWT into routine evaluation may help identify high-risk patients earlier, allow timely escalation of care, and optimize the use of critical care resources. Future multicenter prospective studies with larger sample sizes and serial imaging are needed to validate these findings and to explore their integration into standardized severity scoring systems for dengue infection.
